# Redox regulation of cell proliferation: Bioinformatics and redox proteomics approaches to identify redox-sensitive cell cycle regulators

**DOI:** 10.1016/j.freeradbiomed.2018.03.047

**Published:** 2018-07

**Authors:** Christine H. Foyer, Michael H. Wilson, Megan H. Wright

**Affiliations:** a Centre for Plant Sciences, School of Biology, Faculty of Biological Sciences, University of Leeds, Leeds LS2 9JT, UK; b The Astbury Centre for Structural Molecular Biology, School of Chemistry, University of Leeds, Leeds LS2 9JT, UK

**Keywords:** Cell cycle, Posttranslational modifications, Reactive oxygen species, Retinoblastoma protein, Root apical meristem

## Abstract

Plant stem cells are the foundation of plant growth and development. The balance of quiescence and division is highly regulated, while ensuring that proliferating cells are protected from the adverse effects of environment fluctuations that may damage the genome. Redox regulation is important in both the activation of proliferation and arrest of the cell cycle upon perception of environmental stress. Within this context, reactive oxygen species serve as ‘*pro-life*’ signals with positive roles in the regulation of the cell cycle and survival. However, very little is known about the metabolic mechanisms and redox-sensitive proteins that influence cell cycle progression. We have identified cysteine residues on known cell cycle regulators in Arabidopsis that are potentially accessible, and could play a role in redox regulation, based on secondary structure and solvent accessibility likelihoods for each protein. We propose that redox regulation may function alongside other known posttranslational modifications to control the functions of core cell cycle regulators such as the retinoblastoma protein. Since our current understanding of how redox regulation is involved in cell cycle control is hindered by a lack of knowledge regarding both which residues are important and how modification of those residues alters protein function, we discuss how critical redox modifications can be mapped at the molecular level.

## Highlights

• Reactive oxygen species serve as ‘*pro-life*’ signals with positive roles in the regulation of the cell cycle.
• Increased oxidation of the cytosol and the nucleus occurs during the early stages of the cell cycle.
• Post translational modification of cysteine residues may play a key role in cell cycle regulation.
• We have identified a set of core of cell cycle proteins that have either accessible or potentially exposed cysteines.
• New sensitive methods to characterise the redox proteome will identify redox-regulated cell cycle proteins.

## Graphical abstract

Core cell cycle genes and complexes, showing potentially accessible or exposed cysteines. Coloured boxes show the number of potentially exposed cysteines: Orange, 0 exposed; Green, 1–3 exposed; Blue, 4–12 exposed.

**Figure:**
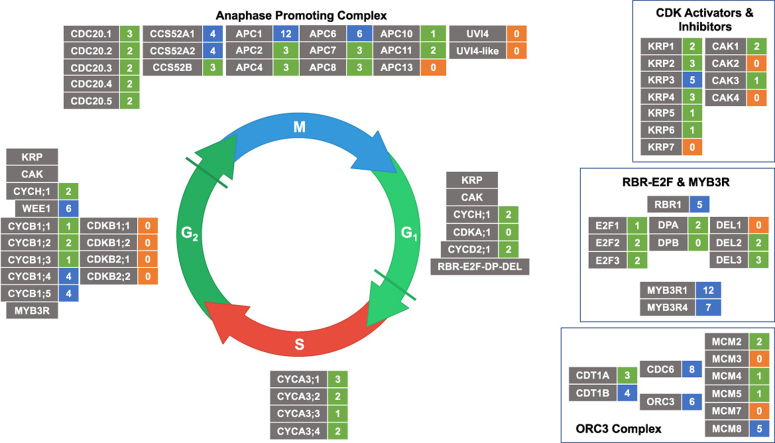

## 1 Introduction

**Fig. 1:**
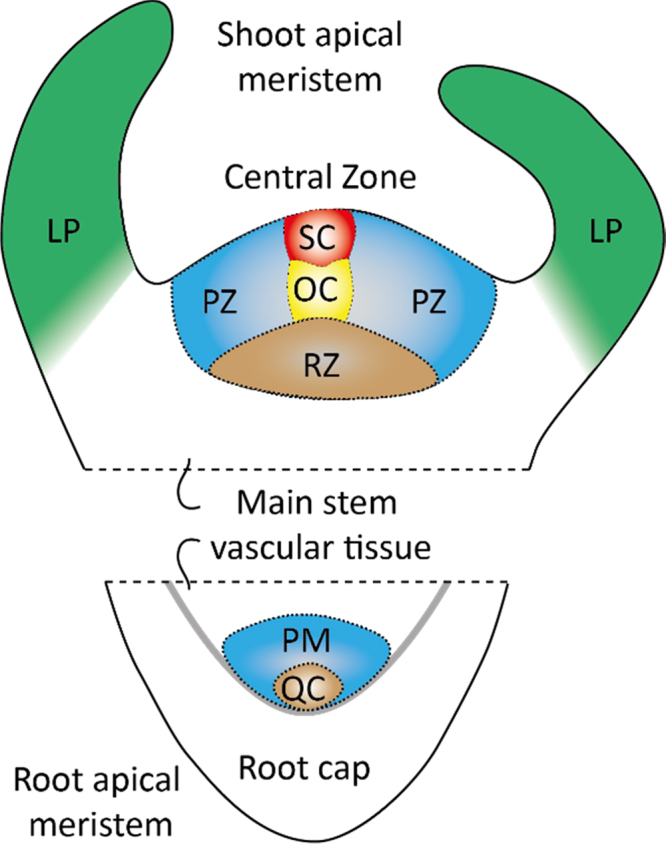
Diagrammatic representation of the structures of the shoot and root apical meristems. LP: leaf primorium; OC: organising centre; PZ: peripheral zone; PM: primary meristem; QC: quiescent centre; PZ: proliferation zone; SC: central zone.

The plant redox signalling network constantly adjusts plant growth and development, as well as metabolism, to prevailing environmental conditions. Within this context reactive oxygen species (ROS) production controls numerous growth and developmental processes by modifying enzyme activity and protein-protein interactions. The accumulation of ROS, either through increased production or regulated decreases in antioxidant capacity, shifts the redox regulatory network to a more oxidising state, in which thiols are oxidised to protein disulfides, sulfenic or sulfinic acid derivatives, as well as glutathionylated and *S*-nitrosylated forms. ROS are well-known regulators of plant growth and cell fate, as well as stress signalling molecules in plants and animals [1], [2], [3]. For example, ROS production is important for tip growth in pollen tubes and root hairs, the respiratory burst oxidase homologue (rboh) NADPH oxidase homologue being required for elongation activities [1]. However, much less is known about the functions of ROS in plant stem cell niches. Plant growth and development is dependent upon the activity of two distinct populations of stem cells that are housed in separate niches, the shoot apical meristem (SAM) that gives rise to all the above ground plant structures and the root apical meristem (RAM) that generates the underground root system, as illustrated in Fig. 1 [4], [5].

Stem cells within these zones are in a process of continuous renewal. They also generate the precursor cells that subsequently divide and differentiate to form all the specialized cells within the plant. A centrally located group of organising stem cells (four in Arabidopsis) within the RAM form the quiescent centre (QC). As in animal stem cell niches, the multipotent stem cells of the plant root QC maintain the surrounding cells in an undifferentiated state [6]. These stem cell ‘initials’ continuously produce daughter cells by asymmetric division. In roots, for example, asymmetric division of the multipotent stem cells generates the columella cells that form the root cap, which protects the RAM [7], [8], [9]. The columella stem cells are maintained in an undifferentiated state by the adjacent QC cells [7].

Upon leaving the meristematic stem cell niche, daughter cells of the multipotent initials undergo subsequent division, elongation, and differentiation to form the differentiated cells of the root, including the vasculature, epidermis and ground tissues [6]. Within the SAM, a central zone (CZ) of about 35 stem cells is localized above the ribmeristem region that provides signals for stem cell maintenance [7], [8]. Progeny of CZ cells move outwards into peripheral zone (PZ) where they undergo division and initiate differentiation into leaf or flower primordia, or downwards into the RM region to differentiate into the stem [8], [10].

The mitochondria in the SAM cells are organized in a perinuclear network suggesting a requirement for mitochondrial functions to drive the cell cycle [11]. The mitochondrial NTPase called APP1 is required for Complex I activity in Arabidopsis. Loss of APP1 functions led to lower levels of ROS accumulation, but increased proliferation of the QC cells and promoted differentiation of distal stem cells [12]. Similarly, the loss of function of an ATP-dependent mitochondrial protease (FTSH4) increased ROS accumulation in the SAM, leading to meristem termination at mildly elevated temperatures [13]. The cellular redox environment required for the cell-to-cell communication that is essential for SAM maintenance can be controlled by regulation of plasmodesmatal, for example by regulation of callose deposition via a plastidial thioredoxin (TRX)m3 [14].

In addition to ROS signalling and regulation of cellular redox state, stem cell identity, cell proliferation and fate are regulated in mammalian cells by a tight control of oxygen availability. The stem cell niche in mammals is maintained at low oxygen partial pressures, physiological hypoxia being central to the integrity of the stem cell niche [15]. Similarly, it is probable that oxidative metabolism is controlled in the SAM and RAM by physiological hypoxia [16]. Hypoxia plays an important role inthe identity and fate of mammalian stem cells [17]. Several lines of evidence suggest that cells in the QC are also maintained in a hypoxic state, and that this may help to preserve genome integrity and pluripotency [18], [19].

In addition to control of oxygen gradients, oxidants such as hydrogen peroxide prime stem cell differentiation in animals and in plants [20], [21], [22], [23], [24], [25], [26]. Cell type-specific transcript profiling has shown that reactive oxygen species (ROS) and ROS-associated genes are expressed in specific zones of the SAM and RAM [27]. ROS and redox components interact with phytohormone signalling to regulate SAM and RAM activities, and different forms of reactive oxygen species (ROS) have been shown to have antagonistic roles in plant stem cell regulation [28]. The UPBEAT1 (UPB1) transcription factor mediates the balance between superoxide and hydrogen peroxide in the root in a way that influences the transition from cell proliferation to cell expansion and differentiation [29]. Accumulation of the superoxide anion in stem cells was shown to activate WUSCHEL, which is the key regulator of plant stem cell maintenance. In contrast, hydrogen peroxide is more abundant in the differentiating peripheral zone, where it promotes stem cell differentiation [29]. Moreover, mutation of an ATP-dependent mitochondrial protease, AtFTSH4, caused increased oxidation of the SAM at high temperatures leading to altered mitochondrial morphology and SAM functions [13]. The regulation of the spatiotemporal patterns of ROS-metabolizing enzymes appears to be important in the orchestration of the balance between superoxide and hydrogen peroxide [26], [29]. Differential and sometimes antagonistic effects of superoxide and hydrogen peroxide in the RAM and SAM have been reported. For example, superoxide was linked to increased expression of the WUSHEL (WUS) transcription factor, which together with CLAVATA peptides determines SAM activity. In contrast, hydrogen peroxide, which accumulated mainly in the peripheral zone of cell differentiation, inhibited WUS expression [26]. Such findings are interesting and intriguing because neither superoxide nor hydrogen peroxide show strong reactivity with other bio-molecules [30]. However, since superoxide and hydrogen peroxide have divergent effects of cell death verses survival signalling in animals, superoxide having an inhibitory effect on cell death pathways and apoptotic signalling [31], it may be that superoxide also promotes cell survival in plants.

Superoxide is a one-electron reduction product of molecular oxygen with no reactivity to most biological molecules. It can, however, interact with nitric oxide (NO) and oxidise ascorbic acid**,** as well as inactivate several enzymes that are important in energy production and amino acid metabolism [30]. Perhaps the most physiology-relevant of these interactions is with the tricarboxylic acid cycle enzyme aconitase. This enzyme is a sensitive target in the mitochondrial matrix. The superoxide-dependent inactivation of this enzyme leads to enhanced glycolysis relative to oxidative phosphorylation in cellular energy generation. While mice lacking Cu, Zn superoxide dismutase have little phenotype, they develop neurological problems and cancers in later life [30]. This finding suggests that superoxide accumulation causes problems only in certain tissues and only at defined stages of animal development [30]. The importance of superoxide accumulation in developing root tips may reside in its effects on intracellular pH rather than its reactivity per se. A decrease in cellular superoxide levels and an increase in hydrogen peroxide results in a shift in the cytosolic pH, making the cellular environment more acid [31].

In plants, extracellular ROS interact with receptor-like kinases (RLKs) in the communication of information perceived in the cellular environment to the interior of the cell. The concurrent initiation of ROS-dependent and ROS-independent signalling linked to RLKs might also be critical in establishing overall cell fate and responses, the RLK-dependent modulation of apoplastic and intracellular conditions being important in regulating ROS perception and signalling [32].

ROS have multi-faceted effects on cell fate. In animals, the concept that ROS are ‘*pro-life*’ signals serving as critical mediators of cytokine signalling with positive roles in survival is supported by a large body of evidence [31]. For example, superoxide and hydrogen peroxide fulfil important roles in proliferative signalling, as well as in triggering programmed cell death, in animals. A burst of ROS stimulate mitogenic pathways in G1 that control CDK activity and the phosphorylation state of the retinoblastoma protein (pRB), thereby regulating S-phase entry. The enhanced oxidation promotes the expression of nuclear factor erythroid-2–related factor 2 (Nrf2). This bZIP transcription factor functions as a master regulator of the cellular response to oxidation [33]. Expression of *Nrf2* re-establishes a reduced intracellular redox state by inducing the expression of cytoprotective genes including those involved in the glutathione and thioredoxin systems. The ROS burst at G1 also triggers the expression of FOXO3, a transcription factor that plays essential roles in cell survival signalling with targets in the cell cycle such as the cyclin-dependent kinase inhibitor (CKI) p27 that is involved in cell cycle withdrawal, as well as defence against oxidative stress [34]. ROS also activate transcription factors such as AP-1 and NF-kB and control a number of early growth-related genes such as *c-fos* and *c-jun* as well as regulating the activities of protein kinases and phosphatases [35], [36]. ROS also have a direct stimulatory effect on tyrosine kinase activity mitogen activated protein kinases (MAPK) like JNK, p38MAPK, and ERK [31]. However, many of the mechanisms that allow ROS to support the *pro-life*’ and survival signalling activities of ROS while also facilitating genetically programmed cell suicide pathways remain to be elucidated in plants and animals.

## 2 Redox regulation of the cell cycle

Cells use proliferative signalling pathways and stress surveillance systems to regulate entry and progress through the cell cycle [37], [38], [39]. Oxidative signalling is important in plants and animals for both the activation of proliferation and arrest of the cell cycle upon perception of environmental or metabolic stresses [40], [41]. Cell cycle progression in animals is driven by an intrinsic redox cycle consisting of reductive and oxidative phases [42]. In this response, growth stimuli induce the cyclin D–CDK4/6 complex, which phosphorylates RB, releasing E2F transcription factors and facilitating the G_1_/S transition. The binding of growth factors, such as epidermal growth factor (EGF) to their receptors (such as EGFR) is promoted by oxidation resulting from ROS accumulation [42], [43], [44]. Redox processes have also been shown to be important in the regulation of RNA polymerase (Pol) III in mammals [45]. In vertebrates, the redox-sensing transcription factor TFIIB-related factor 2 (Brf2), which is a TFIIB-like core transcription factor family member, regulates the formation of a transcriptionally-active pre-initiation complex. This finding suggests direct redox-dependent control of a eukaryotic nuclear RNA polymerase regulates the transcription of genes encoding essential RNAs, a factor that might contribute to the ability of cancer cells to evade ROS-induced cell suicide programs [45].

The A-type CDKs (CDKA) in plants regulate the G1/S and G2/M phase transitions and also function during the S phase, while the B7 1 type CDKs (CDKB) act on the G2/M transition and during the M phase [18], [19]. The D-type CYCs (CYCD) function together with the CDKA during the G1/S transition. The A3 type CYCs (CYCA) operate during the S phase, and with B-type CYCs (CYCB) regulate the G2/M transition. The CYC B1 and CDK1 fractions of mammalian cells that are localised in the mitochondria phosphorylate Complex I subunits during the G2-to-M transition, enhancing mitochondrial respiration and ATP production to drive cell-cycle progression [46]. The concept of redox regulation of cell proliferation is also found in the plant literature [47], [48], [49]. The levels of CYC and CDK transcripts and their activities are changed by oxidative perturbations [27], [35]. Moreover, the regulation of CYC transcription by TEOSINTE BRANCHED1-CYCLOIDEA-PROLIFERATING CELL FACTOR1 (TCP) transcription factors is inhibited by oxidation, possibly via effects on a conserved redox-sensitive cysteine residue that is required for DNA binding [50], [51].

A recent study using a redox-sensitive *in vivo* probe provided the first evidence of a transient oxidation at G1 in the cytosol and nuclei of proliferating cells in the Arabidopsis embryonic root that is perturbed in mutants with low cellular antioxidant levels [52]. This finding supports the concept that "oxidative stress”-sensitive checkpoints are important in the regulation of the cell cycle [38], [39]. The complex redox control of the cell cycle is often explained very simply in terms of a given threshold ROS level required to generate cell proliferation or cell cycle arrest [44]. However, the outcomes of cellular oxidative signalling pathways depend on a number of parameters, principally the chemical nature of ROS form produced (i.e. superoxide, hydrogen peroxide or singlet oxygen) and the nature of the interacting partner (protein thiol, metabolite, lipid or DNA molecule), as well as cell identity. Moreover, the different types of oxidative protein modification (reversible and irreversible) also add a high level of sophistication and specificity to the redox signalling matrix that controls cell proliferation.

Many cellular functions are controlled by redox processes. Local changes in the redox environment mediate the spatio-temporal regulation of protein functions and enzyme activities in a compartment-specific manner. At the molecular level, this is thought to be effected primarily via post translational modification (PTM) of cysteine residues (as discussed below). Redox regulation serves as a crucial PTM and modulator of protein function that is as yet unexplored in relation to the plant cell cycle.

Cell cycle progression is regulated by the activity of cyclin dependent protein kinases (CDKs) and their regulatory partners, which are called cyclins (CYCs) [53], all of which are highly conserved in eukaryotes. The activation of CDKs requires phosphorylation by CDK21 activating kinases (CAKs) and their inactivation involves cyclin dependent kinase inhibitors (CKIs), which are called as Kip-Related Proteins (KRPs) in plants. While the G1/S and G2/M transitions are the major regulatory check points for cell division, meristematic quiescence, dormancy and terminal differentiation in plants are generally characterised by cell cycle arrest at G1 arrest [18]. The cohorts of genes operating at the G1/S- G2/ M phases in plants are regulated by the E2F and the MYB3R transcription factors, which are housed in the multiprotein RBR-MYB3R-E2F complexes that are thought to be related to the DREAM complex in animals [54]. Progression through the G1/S and G2/M phase transitions and S phase is regulated by A-type CDKs (CDKA). For example CDKA;1 is the major RETINOBLATOMA RELATED (RBR) kinase in plants [55]. D-type 2 CYCs (CYCD) operate together with CDKA to regulate the G1/S transition. A3 type CYCs (CYCA) function at S phase. B-type CYCs (CYCB) and CDKs (CDKB) function to regulate the G2/M transition and M. E3 ubiquitin ligases such as the Anaphase Promoting Complex/Cyclosome (APC/C) and Skp1/Cullin/F-box protein (SCF)- related complex, are also important regulators of the cell cycle progress functions to remove cell cycle regulators by proteolysis. The RB protein also shows E2F‐independent functions through binding to other nuclear or extra‐nuclear partners. In mammals, for example, RB cooperates with the MYOD or RUNX2 transcription factors to regulate cell differentiation in an E2F‐independent manner. Moreover, the direct binding of RB to SKP2 suppresses the degradation of p27, attenuating cell cycle progression in an E2F‐independent manner.

Mitogenic signals promote RBR phosphorylation in plants through the action of CDKs in association with D-type cyclins, particularly CYCLIN D3:1 (CYCD3:1). RBR1 is a signal-dependent scaffold protein and a conserved regulator of cell proliferation, differentiation, and stem cell niche maintenance in Arabidopsis [56]. It is regulated by phosphorylation-dependent conformational changes that provide a range of interaction surfaces for diverse complexes and functions [57], [58], [59]. The hypophosphorylated forms of Rb in animals bind to E2F transcription factors during G1 leading to inhibition of cell cycle dependent, E2F-mediated gene expression [49]. Rb preferentially binds to the activating E2F transcription factors, E2F1, E2F2 and E2F3, with their dimerization partners DP1 and DP2 and represses their activity. While E2Fs are not required to drive cell proliferation [60], [61], activating E2Fs promote expression of genes required for DNA synthesis during G1/S. The association between Rb and E2F transcription factors is weakened by sequential phosphorylation, initially by G1-, and then by G1/S- and finally mitosis-specific CDKs. In this way, Rb regulates cell proliferation by decreasing E2F-dependent transcription of cell cycle genes.

Upon phosphorylation, the E2FB transcription factors are released from RBR, enhancing cell cycle gene expression and stimulating cell proliferation, while E2FA remains associated with RBR and maintains meristems through repression of differentiation. The initial phosphorylations are thought to prime further CDK sites for phosphorylation [56]. RBR is phosphorylated on alternative individual sites during G1, leading to mono-phosphorylated RBR forms with distinct properties such as the association with activator E2F1 to regulate checkpoint arrest and apoptosis during DNA damage, or the dissociation from repressor E2F4 to enable the entry into G1/S. The regulatory role of RBR in cell proliferation can be separated from a novel function in safe-guarding genome integrity [62]. Hence there is diversification of the RBR functions through phosphorylation during early G1 phase [55]. We can only speculate that redox regulation of critical cysteines on proteins such as RBR may afford a similar and possibly more important level of control of the cell cycle, in a manner similar to that observed in animals.

## 3 Identification of putative redox-regulated cell cycle proteins in plants

Key questions therefore concern what cell cycle proteins are subject to redox regulation and how redox regulation functions alone or together with other post translational modifications such as protein phosphorylation and ubiquitination to control the cell cycle in plants in response to metabolic, developmental and environmental cues. As a first step to addressing this issue, we downloaded from the Gene Ontology all Arabidopsis gene annotations (http://www.geneontology.org/page/download-annotations) and then extracted the FASTA protein sequence for the representative gene model for each loci identified as cell-cycle related from Ensembl Plants (release-38). We subsequently used solvent accessibility prediction (NetSurfP v1.0 [63]) to identify cysteine residues that are potentially accessible based on secondary structure and solvent accessibility likelihoods for each Arabidopsis protein (Supplemental Table, S1).

**Fig. 2:**
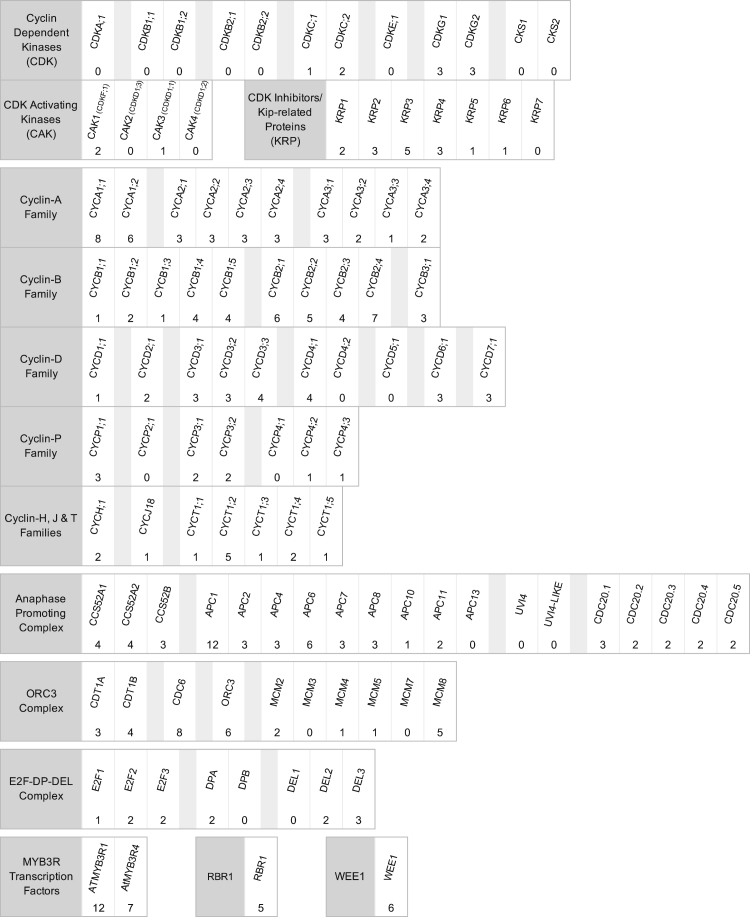
Core cell cycle genes from Arabidopsis, arranged by family and complex. Gene names are presented, AGI codes for loci are in Supplementary table. Numbers represent cysteines potentially surface accessible as calculated by NetSurfP.

**Fig. 3:**
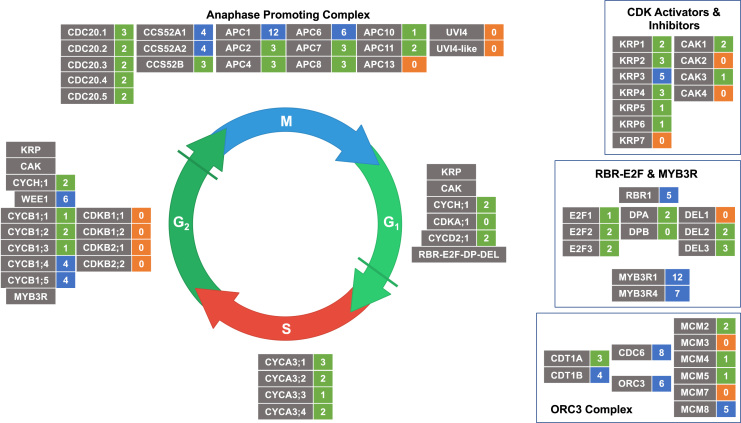
The generalised plant cell cycle, showing the core cell cycle genes and complexes as described in the text, within the context of the cell cycle. Grey boxes, cell cycle gene names, coloured boxes number of potentially exposed cysteines: orange, 0 exposed; green, 1–3 exposed; blue, 4–12 exposed.

From the annotated genes, a restricted set of 108 core of cell cycle proteins [64] was created with some additions (Fig. 2). 22 either had no cysteines or are predicted to have only buried cysteines. Of the remaining 86, the majority (62) had between 1 and 3 accessible residues and the remaining 24 had between 4 and 12 potentially exposed cysteines. This list includes RBR1, WEE1, the MYB3R transcription factors and several members of the cyclin A1, B1, B2 and D families that are key cell cycle regulators, but no CDK, CAK and only one CKI/KRP. Several families of cell-cycle components, including the KRPs the, cyclin D, P and T families, the Anaphase Promoting Complex genes (APC/C), the MCM genes of the ORC complex and the DP and DEL members of the E2F-DP complex, all contain members with several (up to 12) and no exposed cysteine residues, which may be indicative of a sensitive and insensitive population to modulate redox regulation (Fig. 3).

## 4 Mapping redox modifications at the molecular level

Our understanding of how redox regulation is involved in cell cycle control is hindered by a lack of knowledge regarding both which residues are important and how modification of those residues alters protein function. Cysteine redox PTMs are generally formed non-enzymatically via promiscuous reactive electrophilic species (RES) including ROS/RNS/RSS and, whilst widespread redox modification of the proteome, high RES and formation of irreversible oxidations are considered hallmarks of damaging oxidative stress, low levels of RES are crucial for normal cellular function. There is growing appreciation that redox PTMs are site-specific, governed by the microenvironment of cysteine residues [65], and subject to temporal and spatial control, as discussed above.

**Fig. 4:**
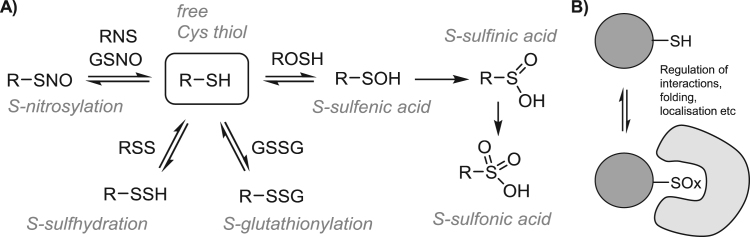
**A)** Cysteine redox modifications. **B)** Redox regulation of protein function.

Cysteine (-SH) is unique amongst amino acid residues in its ability to adopt oxidation states from -2 to + 6 *in vivo*, and thus undergoes a very wide variety of redox-related PTMs [66]. Reversible PTMs include formation of disulfides via reaction with low molecular weight thiols such as glutathione (S-glutathionylation, -SSG), with other proteins (-SSR), or to form *S*-sulfhydrate (-SSH, also known as persulfide); reaction with ROS to generate *S*-sulfenic acid (-SOH), and reaction with RNS in *S*-nitrosylation (-SNO) (Fig. 4). Sulfenic acids (-SOH) can react further to give irreversibly oxidised species, *S*-sulfinic acid (-SO_2_H) and *S*-sulfonic acid (-SO_3_H). Historically this complex array of modifications was difficult to address in a global proteome context and with sufficient sensitivity to detect endogenous oxidative signalling PTMs. However, recent advances in redox proteomic technologies are delivering significant new insights into the scope and biology of redox regulation.

## 5 Tools and platforms for redox proteomics

**Fig. 5:**
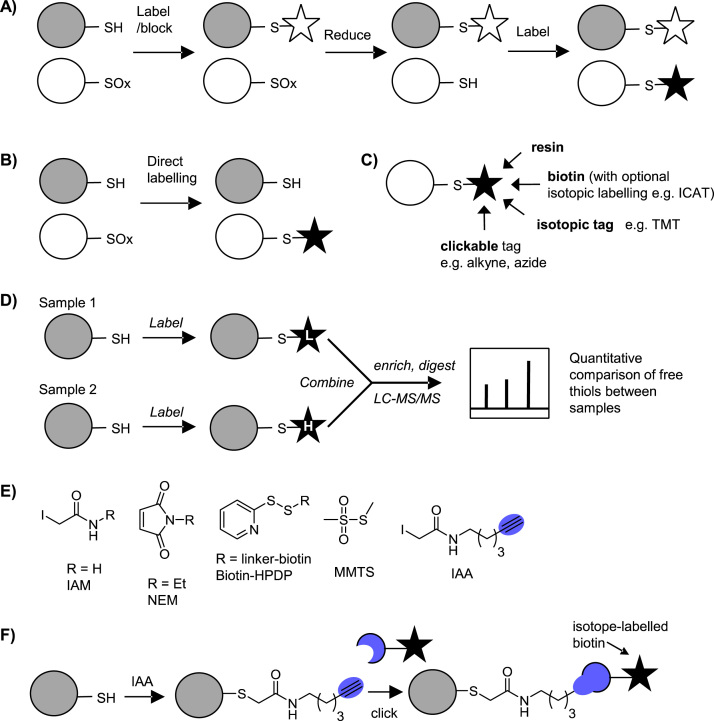
Redox proteomics strategies. Black/white star = label/blocking group. **A)** Indirect approach to detect reversible redox PTMs. **B)** Direct labelling approach. **C)** Labelling of redox PTMs or free thiols via attachment to an affinity tag (e.g. biotin), a pre-tag (e.g. clickable tag), an isotopic label, or a solid support (resin). **D)** Comparative quantitative proteomics: differential labelling of thiols in two samples with heavy and light isotopic reagents for relative quantification of free thiols. **E)** Reagents to label free thiols. **F)** Click-based labelling of thiols with iodoacetamide-alkyne (IAA).

Mass spectrometry (MS)-based proteomics is unrivalled in its ability to identify and characterise large portions of the proteome at the level of individual residues and PTMs. Shotgun proteomics requires sample processing steps including protein denaturation and digest to release peptides for separation and MS detection. Direct MS detection of cysteine redox modifications is therefore not usually possible due to their low abundance and frequent instability. Most redox proteomics strategies therefore exploit the intrinsic chemical reactivity of thiols or cysteine PTMs to introduce labels enabling enrichment (via biotinylation or thiol-reactive resin). Quantification, including of PTM site occupancy, is becoming increasingly important and most redox proteomic approaches lend themselves well to isotope-based comparative quantification, for example exploiting labelling steps to introduce isotopic moieties. Methods can be broadly divided into two categories: those that exploit the intrinsic chemistry of redox PTMs to introduce a label directly at the modification site, and indirect methods that label free thiols then apply selective reduction to release specific cysteine PTM sites for differential labelling (Fig. 5A&B).

Here we review the most common and promising methods for profiling different redox PTMs and their applications in plant biology (summarised in Supplemental Table, S2). For more in depth discussion of how redox PTMs form, the methods used to detect them and considerations of sample preparation, the reader is referred to several excellent recent reviews [67], [68], [69], including Akter et al.’s recent survey of ROS reactivity in plants [70]. The technical details of quantitative proteomic methods, including isobaric tagging (e.g. iTRAQ, TMT), have also been reviewed elsewhere [71].

## 6 Reagents for labelling free thiols

The high nucleophilicity of the free thiol or thiolate relative to other amino acid residues renders it susceptible to selective alkylation with electrophiles, the most commonly used of which are based on iodoacetamide (IAM) and maleimide (e.g. *N*-ethyl maleimide, NEM) (Fig. 5E). MMTS is another common thiol labelling reagent, which reacts via disulfide exchange. Biotinylated versions of IAM and NEM enable direct labelling and enrichment of cysteine-containing proteins for MS analysis, and reagents that incorporate isotope tags have also been developed (see below). These reagents can be used to comparatively label thiols across different conditions (e.g. in the presence and absence of oxidative stress; Fig. 5D). Provided changes in global protein expression are accounted for, this enables indirect determination of sites that are differentially oxidised i.e. decreased detection of a site indicates that it is being blocked by a redox PTM.

A limitation with biotinylation is that bulky reagents may not react efficiently with all thiols and site quantification is not always straightforward. Iodoacetamide-alkyne (IAA; Fig. 5E), developed to profile hyper-reactive cysteines in proteomes [72], contains a small bio-orthogonal tag (such as an azide or alkyne) that can be subsequently ligated to biotin via click chemistry (Fig. 5F). IAA has been applied in a redox proteomics setting to detect changes in thiol oxidation in response to NO donors in lysates [73]. Applying a cleavable and isotopically labelled click reagent [72] enables precise site identification and quantification. The recently reported isotope-incorporating IAA variant [74] should also prove useful in comparing across samples. Another limitation of biotin reagents is that they are not typically cell permeable, so the snapshot of thiols obtained is subject to perturbations that occur upon cell lysis. IAM-based probes are also toxic to cells. To circumvent the toxicity of IAA, versions have been developed which can be uncaged by UV light following cellular uptake [75] and could facilitate *in vivo* detection of cysteine residues.

## 7 Detecting reversible oxidation: biotin-switch and related approaches

Labelling thiols and detecting a reduction in labelling due to oxidation is rather indirect. Differential alkylation, or tag-switch, approaches have been widely applied to label sites of reversible cysteine oxidation (-SOH, -SNO, -SSH/R/G). In the tag-switch approach, alkylation (or blocking) of free thiols is followed by reduction to release reversibly oxidised thiols and then a second labelling step is performed to introduce a means of detecting or enriching modified sites. In the biotin-switch method (BST) [76], biotin is introduced in the second labelling step, commonly via pyridylthiol-biotin (biotin-HPDP), a reagent that forms a reversible disulfide with free thiols and thus enables release of the captured proteins or peptides following enrichment (Fig. 5E). Alternatively, thiols exposed by reduction can be captured on a resin (resin-assisted capture, RAC) [77], labelled with an alternative affinity reagent [78], [79], or alkylated using a different label to that employed in the first blocking step but not enriched [80], [81].

Although biotin is the most common affinity tag due to its extremely high binding affinity with streptavidin, enrichment introduces at least one additional step, resulting in quite lengthy protocols that reduce proteome coverage, and non-specific binding to the resin complicates analysis. To address these challenges, resin-based methods to capture thiols have been reported. Commercially available resin, thiopropyl Sepharose 6B, has been applied to directly capture free thiols via disulfide exchange, followed by on-resin protein digestion and multiplexed isobaric labelling [77] (via iTRAQ - an isotope labelling technique where amines on peptides are labelled with isobaric reagents that can later be distinguished via MS [71]).

**Fig. 6:**
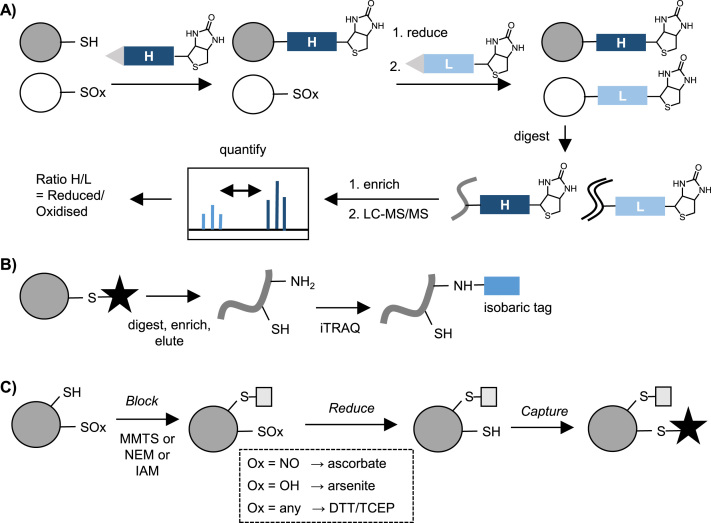
**A)** OxiCAT method of quantifying reversible redox PTMs: %oxidation within a sample. **B)** OxiTRAQ method: quantification is performed via isotopic labelling of peptides on free amine. **C)** Biotin-switch methods. Ox = oxidation. Black star = biotin, resin or other tag.

A popular quantitative method using a tag-switch strategy is OxiCAT, which can be applied to determine PTM site occupancy in a single sample [82]. In OxiCAT both reduced and oxidised cysteines are sequentially captured using isotopically labelled, biotinylated thiol-reactive IAM-based reagents (Fig. 6A). Following digest, biotinylated cysteine-containing peptides are isolated and analysed by LC-MS/MS. Heavy and light cysteine-tagged peptides are distinguishable during MS, generating ratios of oxidised:reduced cysteines. OxiCAT recently revealed >3800 H_2_O_2_-responsive residues in diatoms [83], including 4 with predicted roles in cell cycle control/division and 12 involved in ROS metabolism or redox signalling.

There are several other reported isotopic/isobaric labelling methods for introducing quantification into tag-switch workflows. Like OxiCAT, some introduce isotopic labels at the modification site: these include cysTMT [79] and iodoTMT [84], [85], based on reversible disulfide and irreversible alkylation chemistry respectively. TMT-resin is available for affinity enrichment of labelled peptides. Other methods introduce labels elsewhere on the peptides (usually on free amines). The most widely used of these is OxiTRAQ, where isobaric iTRAQ reagents are applied to peptides after biotin or resin-based enrichment (Fig. 6B). OxiTRAQ has been successfully applied in *Arabidopsis* suspension cells treated with various oxidants or other exogenous agents [86], [87].

Tag-switch methods can be adapted to address specific modifications by using a modification-selective reducing agent (Fig. 6C). An example is detection of *S*-nitrosylation: free thiols are blocked via alkylation, then -SNO modifications reduced to the free thiol using ascorbate, sometimes in conjunction with copper(I) salts; the newly released thiols are labelled via biotinylation for enrichment and detection [76]. Similarly, arsenite was reported to selectively reduce *S*-sulfenic acid [88], although concerns about the compatibility of this PTM with the initial alkylation step means that this strategy has largely been supplanted by direct labelling methods for *S*-sulfenylation (see below). This highlights the main limitation of tag-switch methods: their reliance on the selectivity of alkylating and reducing reagents, which is not complete. In a further example, a recent study investigating cross-reactivity between nitrosothiols and sulfinic acids found that MMTS may release methyl sulfinic acid that could react with *S*-nitrosylation sites and result in slower reduction to the free thiol, such that subsequent detection of -SNO is diminished [89]. Incomplete blocking is also a frequent challenge in tag-switch approaches. Similarly, in most protocols other cysteine PTMs such as *S*-acylation will also be reduced (and then the site alkylated), which can complicate interpretation of results. Nevertheless, these strategies have revealed thousands of potentially redox-sensitive cysteines across diverse proteomes.

## 8 Direct methods for profiling *S*-sulfenylation

**Fig. 7:**
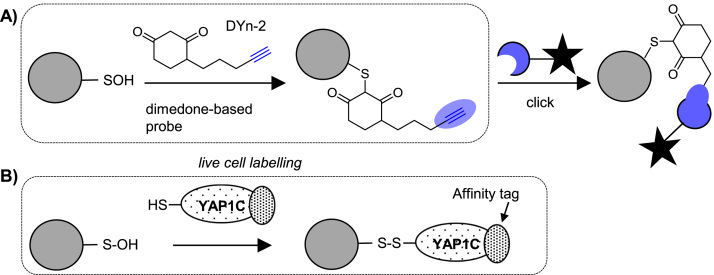
Direct labelling of *S*-sulfenylation. **A)** Dimedone-based probe chemical labelling. **B)** YAP1 genetic probe-based labelling.

*S*-Sulfenylation (-SOH) is a transient PTM with signalling roles, for example in the regulation of protein tyrosine kinases and phosphatases [90], [91], but is not stable under typical MS sample processing conditions [92]. -SOH also cross-reacts with the common thiol blocking reagents, making it difficult to detect via indirect methods (such as the biotin-switch methods described above), although a novel blocking reagent that only reacts with free thiols was recently reported and may prove useful in circumventing this problem [93]. Several reagents for directly labelling -SOH have been developed, mostly inspired by the selective reaction of this PTM with β-dicarbonyls such as dimedone. Antibodies against the dimedone-sulfenic acid adduct are available and have been applied for *S*-sulfenylation profiling in plants [94], but most approaches use biotinylation [95], [96], [97]. Biotinylated reagents [95] are typically applied in cell lysates but cell permeable dimedone-based chemical probes incorporating clickable tags have recently been developed to enable direct labelling of sulfenylated proteins in live cells [90], [98], [99] (Fig. 7A). The small clickable tags allow labelled proteins to be subsequently ligated to biotin after cell lysis. Akter et al. used this method to demonstrate *S*-sulfenylation of 226 proteins in Arabidopsis cell suspensions treated with H_2_O_2_, over half of which had not been previously reported to be modified in plants [100]. Other recent studies have combined clickable dimedone probes with cleavable biotin reagents to detect over 1000 endogenous sulfenylation sites in human cells [99], suggesting that with optimisation of protocols this technology can achieve deep coverage of the endogenous *S*-sulfenome.

A potential limitation of dimedone-based capture is the slow speed of the reaction in comparison to other reactions of sulfenic acids, such as disulfide formation or further oxidation [92], [101], [102]. The Carroll group recently reported several new probes with accelerated reaction rates [101], [103] and demonstrated that structurally different probes label surprisingly distinct sets of endogenous sulfenylated proteins in mammalian cells [103]. Whilst this data suggests that a general probe capable of targeting all sulfenic acids might be challenging to develop, it also means that different subsets of the *S*-sulfenylome, down to specific proteins or protein families, might be targetable with different chemical probes [103].

Bicyclo[6.1.0]nonyne has been reported as alternative chemical probe to trap sulfenic acids, with reaction rates around 100-fold higher than dimedone [102]. Although the known cross-reactivity of thiols with such reagents [104] may present a problem for selective sulfenic acid proteome profiling, subsequent data suggests that this side reaction is not in fact occurring. Thus strained alkynes represent another potential tool for *S*-sulfenylation detection [130].

A completely complementary approach for *S*-sulfenic acid detection uses a cell-based genetic sensor for this PTM, based on the cysteine-rich domain of the yeast transcription factor YAP1, which forms disulfides with *S*-sulfenic acid modifications on its cognate signalling protein. Fusion of the cysteine-rich domain of YAP1 with an affinity tag creates a tool to capture and enrich *S*-sulfenylated proteins *in vivo* (Fig. 7B) [100], [105], [106], [107]. This approach has identified ∼100 sulfenylated proteins in *Arabidopsis thaliana* cells [105].

## 9 Direct probes for *S*-nitrosylation (-SNO)

*S*-Nitrosylation is another labile PTM thought to be mediated primarily by RNS, such as ^•^NO formed by nitric oxide synthetases, and removed via transnitrosylation with small molecular cellular thiols [69], [108]. Whilst the biotin-switch (ascorbate based) method described above [76] is by far the most commonly used, several approaches to directly label or enrich -SNO sites based on their chemistry have also been reported.

**Fig. 8:**
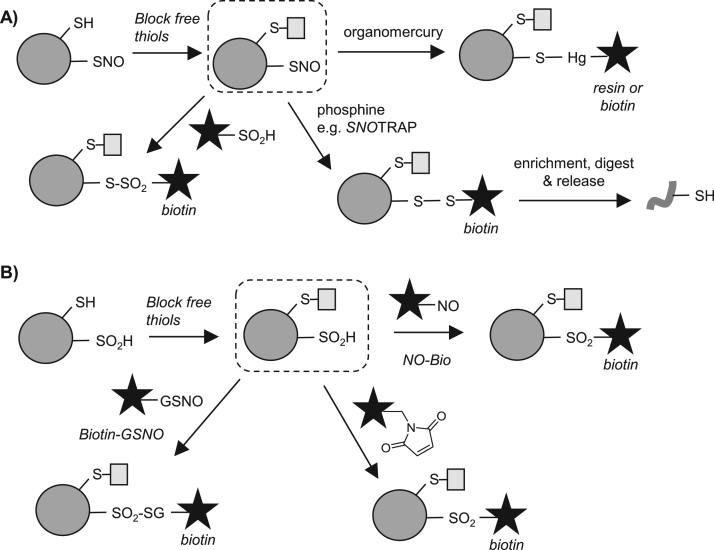
Direct labelling of Cys redox PTMs: **A)***S*-nitrosylation; **B)***S*-sulfinic acid detection methods.

Phenylmercury compounds react with -SNO to generate an S-Hg bond, inspiring the development of mercury-functionalised resins and biotin reagents that can be applied to capture -SNO modifications after blocking of free thiols (Fig. 8A) [109]. Enriched proteins or peptides can then be released via treatment with performic acid, generating a sulfonic acid (-SO_3_H) at the site of modification, which is stable under further sample processing and MS analysis conditions [109]. Another approach used gold nanoparticles to enrich-SNO sites [110], although the selectivity for enrichment of -SNO over other thiol modifications such as disulfides is unclear, which may hinder the utility of this method in complex samples. Phosphine-based reagents (first reported in [111]) can also react with -SNO sites and have enabled -SNO imaging and identification of nitrosylation on select proteins (reviewed in [69]). This chemistry was developed into a global proteomic profiling approach termed *SNO*TRAP (Fig. 8A): free thiols were blocked, then a biotin-incorporating triphenylphosphine thioester used to label -SNO sites, generating a disulfide linkage that could be cleaved following enrichment to release peptides [112]. Phosphine-based probes have known issues in biological samples, such as reaction with disulfides [69], and may require further development to be broadly applicable.

Finally, a recent study investigated the reaction of nitrosothiols and sulfinic acids (-SO_2_H) for mutual detection of these two PTMs [89]. Following blocking of free thiols, biotin-SO_2_H was used to label -SNO, generating a thiosulfonate (Fig. 8A) for enrichment and detection. This method successfully identified nearly 1000 candidate endogenous *S*-nitrosylated proteins in mammalian cells, pinpointing the site of modification for around 100 of these. This study also analysed the relative occupancy of sites.

## 10 Direct probes for *S*-sulfinylation (-SO_2_H) and *S*-sulfonylation (-SO_3_H)

The more highly oxidised and (generally) irreversible redox PTMs of *S*-sulfinylation (-SO_2_H) and *S*-sulfonylation (-SO_3_H) have historically been considered mere markers of oxidative damage, rather than specific controlled signalling PTMs. However, increasing evidence suggests that this may be an over-simplification; for example, transient sulfinylation of peroxiredoxins was shown to be a conserved marker for circadian rhythms across all domains of life [113]. These PTMs cannot be readily analysed by tag-switch approaches because selective reagents for their reduction are not available, and identification of *S*-sulfonylation (-SO_3_H) is thus far limited to direct detection via MS [114] or prior enrichment on polyarginine resin (which also enriches sulfinylated peptides) [115]. However, several direct *S*-sulfinic acid approaches have been reported recently.

Carroll et al. developed novel aryl-nitroso probe NO-Bio to directly capture *S*-sulfinic acids in lysates, following blocking of free thiols (necessary to avoid cross-reactivity) (Fig. 8B) [116]. This represents the first direct labelling method for detecting this redox PTM and should be useful in global MS proteomics studies. Martin et al. have also employed nitroso compounds to detect *S*-sulfinylation, in the reverse of their approach for detecting *S*-nitrosylation via sulfinylation (discussed above) [89]. They applied their Biotin-GSNO reagent (Fig. 8B) to identify endogenous sulfinic acid sites across the human proteome. One limitation of Biotin-GSNO is the relative instability of the reagent, which oxidises over time in aqueous solution [89]. Martin et al. therefore developed an alternative approach, in which, after alkylation of free thiols with IAM, maleimide (NEM-based) reagents react with *S*-sulfinated cysteines to form a sulfone adduct that is stable under acidic conditions [117] (Fig. 8B).

Combined with quantitative MS, these recently developed direct labelling approaches should promote investigation of these understudied PTMs in different biological contexts.

## 11 Detection of sulfhydration/persulfides (-SSH)

**Fig. 9:**
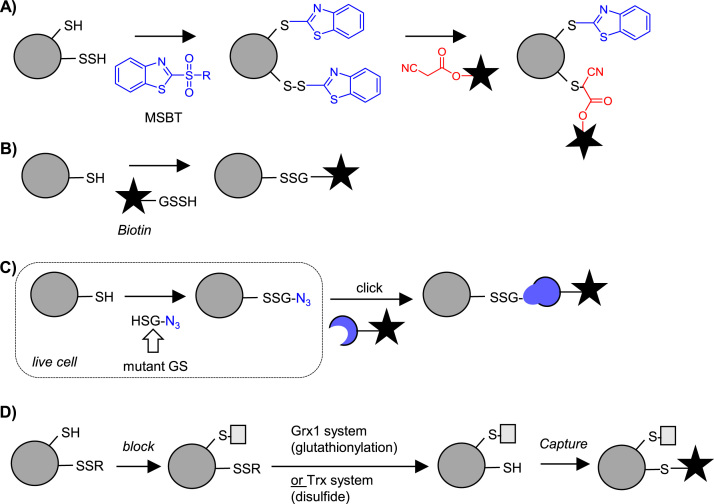
Detection of *S*-sulfhydration, glutathionylation and disulfide modification. **A)** MSBT labelling method for profiling -SSH sites. **B)** Biotinylated-glutathione probes for analysis of cysteines susceptible to glutathionylation. **C)** Metabolic labelling method for the detection of *S*-glutathionylation: Glutathione synthase (GS) produces click-tagged glutathione mimic inside cells, which is incorporated into proteins and can be detected following cell lysis. **D)** Enzymatic methods for detecting substrates of Grx or Trx enzymes.

Identifying sites of sulfhydration (-SSH; also known as persulfides) is challenging due to the similar chemical properties and reactivity of this PTM compared to free thiols. Modified tag-switch approaches have had some success, but site identification is difficult and these methods lack specificity [69]. One approach designed to address some of these issues utilises MSBT to label both thiols and persulfides, followed by selective reduction of the disulfide formed from the latter to generate a free thiol for tagging with, for example, biotin [118]. One limitation of this approach is an inability to discriminate between the newly formed disulfide and other disulfide modifications. To address this and directly label sites of *S*-sulfhydration, Zhang et al. developed chemistry to selectively displace the disulfide that is formed upon MSBT reaction with a CN-biotin reagent, enabling specific enrichment of these sites. (Fig. 9A) [119]. Another approach reacts both thiols and persulfides with IAM-biotin, enriches both sites and then selectively releases the disulfide at the site of persulfide modification from the resin via reduction [120].

## 12 Detection of glutathionylation (-SSG) and disulfides

Protein intra- or intermolecular disulfides are often detected using biotin-switch approaches (see above). The specific modification of cysteine with the low molecular weight thiol glutathione (GSH) and its homodimer disulfide (GSSG), however, can be monitored directly. The first of these direct approaches use biotin-functionalised GSH or GSSG analogues to identify sites susceptible to glutathionylation [121], [122] (Fig. 9B). However, these methods effectively mimic an increase in oxidative stress in cells, rather than profiling endogenous PTMs. To address this, Ahn et al. developed a method where cells are transfected with a mutant glutathione synthetase able to generate *in situ* a GSH analogue incorporating an azide bio-orthogonal tag, which is then metabolically incorporated into cellular proteins (Fig. 9C) [123]. The bio-orthogonal tag is then ligated to biotin using click chemistry. This approach was applied to profile native glutathionylation in cells in response to glucose starvation [124] and shows promise in circumventing the selectivity challenges faced by tag-switch approaches.

The final class of methods that have been developed to address disulfide-type redox PTMs exploit the activity of specific enzyme to classes, including thioredoxin (Trx), which reduces protein disulfides, and glutaredoxin (Grx), which reduces -SSG modifications. Qian, Thrall et al. used an enzymatic version of the tag-switch approach to detect glutathionylation: free thiols were first blocked and then a cocktail containing active Grx1 applied to selectively reduce glutathionylated proteins for subsequent capture on resin and identification (Fig. 9D) [77], [125]. Quantification was introduced via on resin isobaric tagging with either iTRAQ or TMT reagents.

A similar approach has been applied to identify those disulfide-containing proteins that are substrates for the enzyme Trx. Free cysteines are blocked, and then addition of the Trx enzymatic system results in conversion of disulfide-linked proteins to free thiols, which are subsequently biotinylated (Fig. 9D) [126]. An alternative method reported in the same study is to immobilise a mutant Trx on resin and use this to covalently capture Trx substrates upon flow-through. The mutant enzyme lacks one of its two active site cysteines and thus substrate-enzyme intermediates remain trapped until eluted by DTT or another reductant. The “thioredoxome” of the unicellular green alga *Chlamydomonas reinhardtii* was recently extensively characterised using both Trx systems [127].

## 13 Conclusions and perspectives

Recent years have seen rapid evolution of methods to characterise the redox proteome. The key approaches are summarised, along with their advantages and limitations, in Supplemental Table S3. The selectivity of the reagents and enzymes is key to successfully differentiating PTMs across the complex redox landscape, and the chemistry for analysing these continues to develop. This growing intensity of research efforts is promising, as approaches that are sufficiently sensitive to map and (importantly) *quantify* endogenous redox PTMs and *changes* at the residue level are absolutely crucial for analysing subtle changes such as those occurring at different cell cycle stages. Thus the best methods incorporate both quantification and a means of identifying the precise sites of PTM into their workflow. Integrating chemical and enzymatic tools with state-of-the-art mass spectrometry-based instrumentation and optimised workflows also has a large impact. The potential increase in coverage is well-illustrated by the example of OxiCAT: the group who pioneered the method [82] reported quantification of redox status for a few hundred cysteines in the yeast proteome, which was a landmark study at that time [128]; a recent report in yeast identified ~ 4000 sites [129]. Interesting this latter study reached sufficient depth of coverage to quantify the percentage oxidation of cysteines on several cell cycle regulators. OxiCAT is probably currently the best method for obtaining an overview of total reversible oxidation levels across the proteome, as it incorporates quantification and site identification, uses commercially available reagents, and is readily applied across different sample types. However, this method must still be applied in lysates and thus is necessarily subject to artificial changes in redox status that occur upon cell lysis. Alternative approaches - such as methods to label thiols inside live cells - should prove useful in validating data from *in vitro* methods, although few reported studies have directly compared multiple techniques.

In addition to technical developments that advance our ability to interrogate the proteome with existing tools, exciting approaches are emerging to address previously understudied redox PTMs. Examples discussed above include the dimedone-based and more recently developed nucleophilic probes that are able to profile transient *S*-sulfenic acid modifications in live cells, and approaches to tackle PTMs that are difficult to distinguish from other cysteine redox states (e.g. *S*-sulfhydration). We anticipate that method development will also continue to underpin advances in redox biology.

In this review, we have highlighted the central roles of oxygen and redox signalling in regulating cell proliferation, tentatively identified a number of core cell cycle regulators that have the potential to be regulated in this way, and discussed the methods that can be used to analyse and quantify posttranslational redox modifications. Recent progress in increasing the limit and fidelity of detection make studies on the redox regulation of cell cycle control a realistic possibility, at least at the outset in plant cell cultures, where the cell cycle can be synchronised and cell cycle progression can easily be determined, with appropriate amounts of materials harvested for analysis. It will be interesting to determine whether redox processes influence the properties and functions of key cell cycle regulators such as RB and E2Fs, which control DNA replication and mitosis as well as the G_1_/S transition during the cell cycle. The RB family proteins regulate the G_1_/S transition in animals. Inactivation of RB cause oxidative stress by increasing ROS production in mitochondria, where RB is localized. The RB–E2F complex directly suppresses the expression of oxidative metabolism-related enzymes and mitochondrial protein translation genes. It will be intriguing to see if similar mechanisms of redox regulation of cell proliferation are present in plant cells, as well as other redox networks that link cell cycle regulation to metabolism and environmentally sensing. A better understanding of these processes will not only identify the similarities in the redox footprints between plants and animals but also clarify the mechanisms whereby plant stem cells respond to the cellular redox environment. Understanding the functional regulation of redox reactive cysteine residues in cell cycle proteins, and their interactions with mitochondrial and plastid signalling networks, will lead to a step change in our appreciation of how cell metabolism uses the redox reactions that harness energy for life to control cell proliferation and survival in plants and animals.
