# Supplementary material for: Redox regulation of cell proliferation: Bioinformatics and redox proteomics approaches to identify redox-sensitive cell cycle regulators
Source: Free Radic Biol Med. 2018 Jul;122:137–49. doi: 10.1016/j.freeradbiomed.2018.03.047 (PMC6146653; doi:10.1016/j.freeradbiomed.2018.03.047)
Supplement: Supplementary file 1 — Supplementary material [file mmc1.pdf]

Supplemental Table S3. Comparison of redox proteomics methodologies.

Methodology type	General comments on approach	Details	Advantages	Additional limitations (beyond general approach)
Reversible oxidation (-SOx)				
Indirect: tag switch	- lysates only<br>- indirect method & multistep	**Classic BST:**<br>- Block (IAM, MMTS or NEM)<br>- reduce (DTT/TCEP)<br>- label (biotin-HPDP/NEM)<br>- enrich on resin	- In principle widely applicable once optimised.	- Additional steps required for quantification or use label-free.<br>- Site ID indirect.<br>- Many blocking reagents may crossreact with *S*-sulfenic acid.
		Variant with **CysTMT** labelling [1]<br>Variant with **IodoTMT** labelling [2]	Site ID (direct) and quant. (up to 6 samples).	- No enrichment<br>- High analytical requirements for TMT.
		**OxiTRAQ** (block, reduce, label, enrich, iTRAQ labelling of eluted peptides) [3]	Site ID (indirect) and quantification.	Protocol with large number of steps.
		**OxiCAT** (block, reduce, label with iCAT reagents, enrich, cleave from resin with acid) [4]	- Site ID (direct) and quant.<br>- Robust, widely used.	Only 2 samples can be compared (c.f. TMT).
		**Resin-assisted enrichment** variants (“label” step replaced with direct resin capture) [5]	- Site ID (indirect).<br>- Eliminates a step.	Additional steps required for quantification or use label-free.
Indirect: detect absence of labelling	- cells possible	In cell labelling of free thiols with **iodoacetamide-alkyne** or similar e.g. caged variant. Click on biotin [6, 7]	- Site ID (direct for free thiol) and quant. possible with isotope-encoded reagent.	- indirect detection of oxidised thiols via loss of labelling
S-nitrosylation (-SNO)				
Indirect: tag switch	- lysates only<br>- ascorbate reduction not completely selective<br>- indirect method & multi-step	**Classic BST for NO:** [8]<br>- Block (IAM, NEM or MMTS)<br>- ascorbate reduction<br>- label (biotin-HPDP/NEM)	- In principle widely applicable once optimised.	- Additional steps required for quantification or use label-free.<br>- Site ID indirect or not always possible.
		Variant with **ICAT** at label step.	- Quantification introduced via isotopic ICAT label.<br>- Site ID via cleavable ICAT.	
Semi-direct: block, direct label	- lysates only<br>- requires blocking of free thiols before direct labelling<br>- advantage: no reduction step required	**Organomercury** reagents / resin	- site ID possible	- indirect site ID (elute thiol from resin)
		Phosphine-based reagents e.g. ***SNO*TRAP** [9]	- site ID possible	- reagents may react with disulfides too<br>- indirect site ID (elute thiol from resin)
		***S*-sulfinic acid** reagents [10]	- site ID possible	- reagent has stability issues

Methodology type	General comments on approach	Details	Advantages	Additional limitations (beyond general approach)
S-sulfenylation				
Indirect: tag-switch	- lysates only<br>- indirect method & multistep	**Tag-switch**<br>- block (IAM, MMTS, NEM)<br>- reduce -SOH (arsenite)<br>- label (biotin-HPDP/NEM etc)		Selectivity problems: -SOH is susceptible to initial blocking step
Direct labelling	- lysates<br>- cell permeable tool has been applied for imaging	**Strained alkyne (BCN)** [11, 12]		Possible cross-reactivity of BCN reagent with thiols?
Direct labelling	Advantages:<br>- *in vivo* possible<br><br>Limitations:<br>- most commonly used reagents (i.e. dimedone-based) react slowly. New reagents in development (see main text).	**Dimedone-based biotin probes** [13]		- only in lysates.<br>- site ID not straightforward<br>- additional steps required for quantification or use label-free.
		**Dimedone-based click probes** [14]<br>Label, click chemistry, enrich (via biotin). Quantification can be introduced via isotope-labelled click reagents.	- Can be applied *in vivo*<br>- Site ID (direct) and quant. possible via use of cleavable click reagents.	- additional step required (click chemistry)
		**YAP1 *in vivo* genetically encoded probe** [15]	- Direct labelling in cells.<br>- Genetically-encoded protein-based probes can be localised to specific organelles.	- requires genetic manipulation – only possible for some sample types.<br>- system could perturb local redox states<br>- limited dose and temporal control
S-sulfinylation (-SO_2H)				
Semi-direct: block then direct labelling	- lysates only<br>- requires blocking of free thiols before labelling<br>- advantage: no reduction step required	**NO-Bio** [16]		- not yet applied for proteomic analysis
		**Biotin-GSNO** [10]		- reagent has stability issues
		**NEM-based** [17]	- reagent is stable	- labelled adduct only stable under acidic conditions

Methodology type	General comments on approach	Details	Advantages	Additional limitations (beyond general approach)
S-sulfhydration				
Indirect: tag-switch	- lysates only	**Tag-switch.** Block free thiols (MMTS), react -SSH sites with biotin-HPDP.		unclear if MMTS is truly selective for -SH over -SSH
Semi-direct	- lysates only (possibly parts of workflows adaptable to cells?)	**Reduction tag-switch.** Alkylation of -SH and -SSH with IAA or MSBT, followed by cleavage (via reduction) and labelling of former -SSH site with biotin. Enrichment and elution. [18]	- site ID (indirect)	Selectivity problem: other disulfides (-SSR) also released by reduction and labelled.
		Alkylation of both free thiols and persulfides with **IAM-biotin**, enrichment and selective elution of former -SSH site from resin. [19]	- more streamlined than reduction tag-switch method<br>- site ID (indirect)	- selectivity problems if peptide also contains a disulfide site
		**Labelling tag-switch**. Alkylation of both free thiols and persulfides with **MSBT**, followed by selective reaction of former -SSH site with **CN-biotin** reagent. Enrichment and elution. [20]	- site ID<br>- overcomes selectivity problem of reduction tag-switch method	
S-glutathionylation and disulfides				
Direct	- in cells	**Biotin-GSH or -GSSH** added to cells. Lysis and enrichment of sites. [21, 22]	- can be performed in cells<br>- site identification	- not measuring endogenous level (mimics increase in oxidative stress)
		**Metabolic *in situ* Azido-GSH generation**. [23]	- performed in cells<br>- enables endogenous measurement	- requires expression mutant enzyme in cells
Indirect	- in lysates<br>- relies on complete blocking	**Enzymatic tag-switch** for -SSG. Blocking of free thiols; Grx1 enzyme system added to reduce -SSG; newly released free thiols captured on resin or biotinylated. [5]	- enzymes are highly specific and operate under physiological conditions	- only identifies Grx/Trx-susceptible modified proteins (subset)<br>- multi-step
		**Enzymatic tag-switch** for -SSR. Blocking of free thiols; Trx enzyme system added to reduce -SSR; newly released free thiols captured on resin or biotinylated. [24]		
		**Enzymatic capture** for -SSR. Blocking of free thiols; capture of Trx-susceptible disulfides via immobilised mutant Trx; elution with DTT. [25]	- enzymes are highly specific and operate under physiological conditions<br>- fewer steps	- only identifies Trx-susceptible modified proteins (subset)

References

[1] Murray, C. I.; Uhrigshardt, H.; O'Meally, R. N.; Cole, R. N.; Van Eyk, J. E. Identification and quantification of S-nitrosylation by cysteine reactive tandem mass tag switch assay. *Mol Cell Proteomics* **11:**M111 013441; 2012.

[2] Qu, Z.; Meng, F.; Bomgarden, R. D.; Viner, R. I.; Li, J.; Rogers, J. C.; Cheng, J.; Greenlief, C. M.; Cui, J.; Lubahn, D. B.; Sun, G. Y.; Gu, Z. Proteomic quantification and site-mapping of S-nitrosylated proteins using isobaric iodoTMT reagents. *J Proteome Res* **13:**3200-3211; 2014.

[3] Liu, P.; Zhang, H.; Wang, H.; Xia, Y. Identification of redox-sensitive cysteines in the Arabidopsis proteome using OxiTRAQ, a quantitative redox proteomics method. *Proteomics* **14:**750-762; 2014.

[4] Leichert, L. I.; Gehrke, F.; Gudiseva, H. V.; Blackwell, T.; Ilbert, M.; Walker, A. K.; Strahler, J. R.; Andrews, P. C.; Jakob, U. Quantifying changes in the thiol redox proteome upon oxidative stress in vivo. *Proc Natl Acad Sci U S A* **105:**8197-8202; 2008.

[5] Su, D.; Gaffrey, M. J.; Guo, J.; Hatchell, K. E.; Chu, R. K.; Clauss, T. R.; Aldrich, J. T.; Wu, S.; Purvine, S.; Camp, D. G.; Smith, R. D.; Thrall, B. D.; Qian, W. J. Proteomic identification and quantification of S-glutathionylation in mouse macrophages using resin-assisted enrichment and isobaric labeling. *Free Radic Biol Med* **67:**460-470; 2014.

[6] Abo, M.; Weerapana, E. A Caged Electrophilic Probe for Global Analysis of Cysteine Reactivity in Living Cells. *J Am Chem Soc* **137:**7087-7090; 2015.

[7] Zhou, Y.; Wynia-Smith, S. L.; Couvertier, S. M.; Kalous, K. S.; Marletta, M. A.; Smith, B. C.; Weerapana, E. Chemoproteomic Strategy to Quantitatively Monitor Transnitrosation Uncovers Functionally Relevant S-Nitrosation Sites on Cathepsin D and HADH2. *Cell Chem Biol* **23:**727-737; 2016.

[8] Jaffrey, S. R.; Snyder, S. H. The biotin switch method for the detection of S-nitrosylated proteins. *Sci STKE* **2001:**pl1; 2001.

[9] Seneviratne, U.; Nott, A.; Bhat, V. B.; Ravindra, K. C.; Wishnok, J. S.; Tsai, L. H.; Tannenbaum, S. R. S-nitrosation of proteins relevant to Alzheimer's disease during early stages of neurodegeneration. *Proc Natl Acad Sci U S A* **113:**4152-4157; 2016.

[10] Majmudar, J. D.; Konopko, A. M.; Labby, K. J.; Tom, C. T.; Crellin, J. E.; Prakash, A.; Martin, B. R. Harnessing Redox Cross-Reactivity To Profile Distinct Cysteine Modifications. *J Am Chem Soc* **138:**1852-1859; 2016.

[11] Poole, T. H.; Reisz, J. A.; Zhao, W.; Poole, L. B.; Furdui, C. M.; King, S. B. Strained cycloalkynes as new protein sulfenic acid traps. *J Am Chem Soc* **136:**6167-6170; 2014.

[12] McGarry, D. J.; Shchepinova, M. M.; Lilla, S.; Hartley, R. C.; Olson, M. F. A Cell-Permeable Biscyclooctyne As a Novel Probe for the Identification of Protein Sulfenic Acids. *ACS Chem Biol* **11:**3300-3304; 2016.

[13] Poole, L. B.; Klomsiri, C.; Knaggs, S. A.; Furdui, C. M.; Nelson, K. J.; Thomas, M. J.; Fetrow, J. S.; Daniel, L. W.; King, S. B. Fluorescent and affinity-based tools to detect cysteine sulfenic acid formation in proteins. *Bioconjug Chem* **18:**2004-2017; 2007.

[14] Yang, J.; Gupta, V.; Carroll, K. S.; Liebler, D. C. Site-specific mapping and quantification of protein S-sulphenylation in cells. *Nat Commun* **5:**4776; 2014.

[15] Takanishi, C. L.; Ma, L. H.; Wood, M. J. A genetically encoded probe for cysteine sulfenic acid protein modification in vivo. *Biochemistry* **46:**14725-14732; 2007.

[16] Lo Conte, M.; Lin, J.; Wilson, M. A.; Carroll, K. S. A Chemical Approach for the Detection of Protein Sulfinylation. *ACS Chem Biol* **10:**1825-1830; 2015.

[17] Kuo, Y. H.; Konopko, A. M.; Borotto, N. B.; Majmudar, J. D.; Haynes, S. E.; Martin, B. R. Profiling Protein S-Sulfination with Maleimide-Linked Probes. *Chembiochem* **18:**2028-2032; 2017.
[18] Wedmann, R.; Onderka, C.; Wei, S.; Szijarto, I. A.; Miljkovic, J. L.; Mitrovic, A.; Lange, M.; Savitsky, S.; Yadav, P. K.; Torregrossa, R.; Harrer, E. G.; Harrer, T.; Ishii, I.; Gollasch, M.; Wood, M. E.; Galardon, E.; Xian, M.; Whiteman, M.; Banerjee, R.; Filipovic, M. R. Improved tag-switch method reveals that thioredoxin acts as depersulfidase and controls the intracellular levels of protein persulfidation. *Chem Sci* **7:**3414-3426; 2016.
[19] Doka, E.; Pader, I.; Biro, A.; Johansson, K.; Cheng, Q.; Ballago, K.; Prigge, J. R.; Pastor-Flores, D.; Dick, T. P.; Schmidt, E. E.; Arner, E. S.; Nagy, P. A novel persulfide detection method reveals protein persulfide- and polysulfide-reducing functions of thioredoxin and glutathione systems. *Sci Adv* **2:**e1500968; 2016.
[20] Zhang, D.; Macinkovic, I.; Devarie-Baez, N. O.; Pan, J.; Park, C. M.; Carroll, K. S.; Filipovic, M. R.; Xian, M. Detection of protein S-sulfhydration by a tag-switch technique. *Angew Chem Int Ed Engl* **53:**575-581; 2014.
[21] Sullivan, D. M.; Wehr, N. B.; Fergusson, M. M.; Levine, R. L.; Finkel, T. Identification of oxidant-sensitive proteins: TNF-alpha induces protein glutathiolation. *Biochemistry* **39:**11121-11128; 2000.
[22] Brennan, J. P.; Miller, J. I.; Fuller, W.; Wait, R.; Begum, S.; Dunn, M. J.; Eaton, P. The utility of N,N-biotinyl glutathione disulfide in the study of protein S-glutathiolation. *Mol Cell Proteomics* **5:**215-225; 2006.
[23] Samarasinghe, K. T.; Munkanatta Godage, D. N.; VanHecke, G. C.; Ahn, Y. H. Metabolic synthesis of clickable glutathione for chemoselective detection of glutathionylation. *J Am Chem Soc* **136:**11566-11569; 2014.
[24] Marchand, C.; Le Marechal, P.; Meyer, Y.; Decottignies, P. Comparative proteomic approaches for the isolation of proteins interacting with thioredoxin. *Proteomics* **6:**6528-6537; 2006.
[25] Perez-Perez, M. E.; Mauries, A.; Maes, A.; Tourasse, N. J.; Hamon, M.; Lemaire, S. D.; Marchand, C. H. The deep thioredoxome in Chlamydomonas reinhardtii: new insights into redox regulation. *Mol Plant*; 2017.
