# Supplementary material for: Redox regulation of cell proliferation: Bioinformatics and redox proteomics approaches to identify redox-sensitive cell cycle regulators
Source: Free Radic Biol Med. 2018 Jul;122:137–49. doi: 10.1016/j.freeradbiomed.2018.03.047 (PMC6146653; doi:10.1016/j.freeradbiomed.2018.03.047)
Supplement: Supplementary file 3 — Supplementary material [file mmc3.pdf]

Global redox proteomics studies in plants and other photosynthetic organisms: *S*-Nitrosylation * = endogenous level detection

Biological system	Methodology	Quantification and site identification	Reference
***S*-nitrosylation**			
Arabidopsis cell extract treated (GSNO); leaves treated with NO gas	BST-NO-standard (MMTS; ascorbate; biotin-HPDP)	protein ID only	Lindermayr et al. 2005 [1]
Arabidopsis leaf extract treated with GSNO	BST-NO-standard	protein ID only	Romero-Puertas et al. 2008 [2]
Kalanchoe pinnata extract treated with GSNO	BST-NO-standard	protein ID only	Abat et al. 2008 [3]
Brassica juncea extract treated with GSNO	BST-NO-standard	protein ID only	Abat et al. 2009 [4]
Arabidopsis mitochondria treated with GSNO	BST-NO-standard	protein ID only	Palmieri et al. 2010 [5]
Antiaris toxicaria treated with NO gas	BST-NO-standard	protein ID only	Bai et al. 2011 [6]
Arabidopsis suspension cells *	BST (MMTS; ascorbate; ICAT)	site ID; quantification (ICAT) for response to salt stress	Fares et al. 2011 [7]
Potato extract treated with GSNO	BST-NO-standard	protein ID only	Kato et al. 2012 [8]
Oryza sativa rice: wild-type and noe1 mutant rice plants *	BST-NO-standard	protein ID only	Lin et al. 2012 [9]
Pea *(Pisum sativum)* plant peroxisomes *	BST-NO-standard	protein ID only	Ortega-Galisteo et al. 2012 [10]
N. tabacum cv. Xanthi cell suspensions *	BST-NO-standard	protein ID only	Astier et al. 2012 [11]
Pea *(Pisum sativum)* plant mitochondria *	BST-NO-standard	protein ID only	Camejo et al. 2012 [12]
Citrus aurantium L.; NaCl stress *	BST-NO-standard	protein ID only	Tanou et al. 2012 [13] and 2014 [14]
Brassica juncea subject to cold stress *	BST-NO-standard	protein ID only	Sehrawat et al. 2013 [15]
Brassica juncea apoplast extracts treated with GSNO	BST-NO-standard	protein ID only	Sehrawat et al. 2014 [16]
Arabidopsis plants *	BST (MMTS; ascorbate; ICAT)	site ID; quantification (ICAT) for response to cold stress	Puyaubert et al. 2014 [17]
Poplar exposed to ozone *	BST (NEM; ascorbate; biotin-HPDP)	protein ID only	Vanzo et al. 2014 [18]
Chlamydomonas reinhardtii cells treated with RES	BST (IAM & NEM; ascorbate; biotin-HPDP)	site ID	Morisse et al. 2014 [19]
Arabidopsis nuclei treated with GSNO	BST-NO-standard	protein ID only	Chaki et al. 2015 [20]
Poplar and non-isoprene-emitting variant exposed to ozone *	BST (NEM; ascorbate; biotin-HPDP)	protein ID only	Vanzo et al. 2016 [21]
Chlorella vulgaris cells grown in presence and absence of nitrate *	BST-NO-standard	protein ID only	Henard et al. 2017 [22]
Arabidopsis *	BST-NO-standard	protein ID only	Hu et al. 2015 [23]
	BST (IAM; ascorbate; biotin-NEM)	site ID	

Total reversible oxidation

Biological system	Methodology	Quantification and site identification	Reference
**Total reversible oxidation**	**Indirect switch approaches**		
Tomato plants infected with *Pseudomonas syringae* *	Blocking (IAM); reduction (TCEP); CysTMT labelling.	site ID	Parker et al. 2012 [24]<br>Balmant et al. 2015 [25]
Arabidopsis suspension cells treated with bicarbonate *	Blocking (NEM), reduction (TCEP) and IodoTMT labelling. (no enrichment)	site ID and quant. via TMT label; quant. abundance via iTRAQ	Yin et al. 2017 [26]
Cyanobacteria in light and dark conditions *	Blocking (NEM), reduction (DTT) and resin-based enrichment. TMT labelling on-resin.	site ID via peptide enrichment; TMT for quant. % oxidation.	Guo et al. 2015 [27]
Arabidopsis suspension cells * plus treated with H_2O_2	OxiTRAQ. Blocking (NEM), reduction (DTT) and resin-enrichment. iTRAQ labelling eluted peptides.	site ID	Liu et al. 2014 [28]
Diatom extracts * plus treated with H_2O_2	OxiCAT. Alkylation (IAM-L-biotin); reduction (TCEP); alkylation (IAM-H-biotin)	site ID; %oxidation; quantified change in %oxidation upon H_2O_2	Rosenwasser et al. 2014 [29]
Brassica napus guard-cell response to abscisic acid (ABA) or methyl jasmonate (MeJA) *	DIGE and OxiCAT compared. (IAM; TCEP; label)	site ID for iCAT	Zhu et al. 2014 [30]
Arabidopsis cell suspension * plus treated with salicylate or flg22	OxiTRAQ. Blocking (NEM), reduction (DTT), Biotin-HPDP. iTRAQ labelling of eluted peptides.	site ID; iTRAQ to correct for protein abundance changes	Liu et al. 2015 [31]
C. reinhardtii algae and *Arabidopsis*. *C. reinhardtii* treated with H_2O_2	Blocking (IAM), reduction (DTT) and resin-based enrichment. DTT elution.	site ID; label-free quantification	Slade et al. 2015 [32]
Arabidopsis chloroplasts * plus treated with H_2O_2	Blocking (NEM); reduction (DTT); label with biotin-NEM.	protein ID only	Muthuramalingam et al. 2013 [33]

Other redox PTMs

Biological system	Methodology	Quantification and site identification	Reference
***S*-sulfenylation**			
Arabidopsis cells treated with H_2O_2	Dimedone-based DYn-2 *in vivo*. Click to biotin.	protein ID only	Akter et al. 2015 [34]
Arabidopsis cells treated with H_2O_2	YAP1 *in vivo* trapping	protein ID only	Waszczak et al. 2014 [35]
Arabidopsis cells treated with H_2O_2	Dimedone antibody	protein ID only	Akter et al. 2017 [36]
***S*-Glutathionylation**			
Arabidopsis cells	Treatment with Biotin-GEE	protein ID only	Ito et al. 2003 [37]
Arabidopsis cells	Treatment with Biotin-GSSG	protein ID only	Dixon et al. 2005 [38]
C. reinhardtii algae	Treatment with Biotin-GSSG	site ID	Zaffagnini et al. 2012 [39]
***S*-Sulfhydration**			
Arabidopsis leaf extract *	Block (MMTS); biotin-HPDP	protein ID only	Aroca et al. 2015 [40]
Arabidopsis and DES1-(H_2S production) defective mutant plants *	MSTB labelling, CN-biotin displacement	protein ID; TMT quantification	Aroca et al. 2017 [41]

Trx- and Grx-susceptible protein disulfide PTMs were recently reviewed by Bykova and Rampitsch, 2013 [42]

References

[1] Lindermayr, C.; Saalbach, G.; Durner, J. Proteomic identification of S-nitrosylated proteins in Arabidopsis. *Plant Physiol* **137:**921-930; 2005.
[2] Romero-Puertas, M. C.; Campostrini, N.; Matte, A.; Righetti, P. G.; Perazzolli, M.; Zolla, L.; Roepstorff, P.; Delledonne, M. Proteomic analysis of S-nitrosylated proteins in Arabidopsis thaliana undergoing hypersensitive response. *Proteomics* **8:**1459-1469; 2008.
[3] Abat, J. K.; Mattoo, A. K.; Deswal, R. S-nitrosylated proteins of a medicinal CAM plant Kalanchoe pinnata- ribulose-1,5-bisphosphate carboxylase/oxygenase activity targeted for inhibition. *FEBS J* **275:**2862-2872; 2008.
[4] Abat, J. K.; Deswal, R. Differential modulation of S-nitrosoproteome of Brassica juncea by low temperature: change in S-nitrosylation of Rubisco is responsible for the inactivation of its carboxylase activity. *Proteomics* **9:**4368-4380; 2009.
[5] Palmieri, M. C.; Lindermayr, C.; Bauwe, H.; Steinhauser, C.; Durner, J. Regulation of plant glycine decarboxylase by s-nitrosylation and glutathionylation. *Plant Physiol* **152:**1514-1528; 2010.
[6] Bai, X.; Yang, L.; Tian, M.; Chen, J.; Shi, J.; Yang, Y.; Hu, X. Nitric oxide enhances desiccation tolerance of recalcitrant Antiaris toxicaria seeds via protein S-nitrosylation and carbonylation. *PLoS One* **6:**e20714; 2011.
[7] Fares, A.; Rossignol, M.; Peltier, J. B. Proteomics investigation of endogenous S-nitrosylation in Arabidopsis. *Biochem Biophys Res Commun* **416:**331-336; 2011.
[8] Kato, H.; Takemoto, D.; Kawakita, K. Proteomic analysis of S-nitrosylated proteins in potato plant. *Physiol Plant* **148:**371-386; 2013.
[9] Lin, A.; Wang, Y.; Tang, J.; Xue, P.; Li, C.; Liu, L.; Hu, B.; Yang, F.; Loake, G. J.; Chu, C. Nitric oxide and protein S-nitrosylation are integral to hydrogen peroxide-induced leaf cell death in rice. *Plant Physiol* **158:**451-464; 2012.
[10] Ortega-Galisteo, A. P.; Rodriguez-Serrano, M.; Pazmino, D. M.; Gupta, D. K.; Sandalio, L. M.; Romero-Puertas, M. C. S-Nitrosylated proteins in pea (Pisum sativum L.) leaf peroxisomes: changes under abiotic stress. *J Exp Bot* **63:**2089-2103; 2012.
[11] Astier, J.; Besson-Bard, A.; Lamotte, O.; Bertoldo, J.; Bourque, S.; Terenzi, H.; Wendehenne, D. Nitric oxide inhibits the ATPase activity of the chaperone-like AAA+ ATPase CDC48, a target for S-nitrosylation in cryptogein signalling in tobacco cells. *Biochem J* **447:**249-260; 2012.
[12] Camejo, D.; Romero-Puertas Mdel, C.; Rodriguez-Serrano, M.; Sandalio, L. M.; Lazaro, J. J.; Jimenez, A.; Sevilla, F. Salinity-induced changes in S-nitrosylation of pea mitochondrial proteins. *J Proteomics* **79:**87-99; 2013.
[13] Tanou, G.; Filippou, P.; Belghazi, M.; Job, D.; Diamantidis, G.; Fotopoulos, V.; Molassiotis, A. Oxidative and nitrosative-based signaling and associated post-translational modifications orchestrate the acclimation of citrus plants to salinity stress. *Plant J* **72:**585-599; 2012.
[14] Tanou, G.; Ziogas, V.; Belghazi, M.; Christou, A.; Filippou, P.; Job, D.; Fotopoulos, V.; Molassiotis, A. Polyamines reprogram oxidative and nitrosative status and the proteome of citrus plants exposed to salinity stress. *Plant Cell Environ* **37:**864-885; 2014.
[15] Sehrawat, A.; Abat, J. K.; Deswal, R. RuBisCO depletion improved proteome coverage of cold responsive S-nitrosylated targets in Brassica juncea. *Front Plant Sci* **4:**342; 2013.
[16] Sehrawat, A.; Deswal, R. S-nitrosylation analysis in Brassica juncea apoplast highlights the importance of nitric oxide in cold-stress signaling. *J Proteome Res* **13:**2599-2619; 2014.
[17] Puyaubert, J.; Fares, A.; Reze, N.; Peltier, J. B.; Baudouin, E. Identification of endogenously S-nitrosylated proteins in Arabidopsis plantlets: effect of cold stress on cysteine nitrosylation level. *Plant Sci* **215-216:**150-156; 2014.
[18] Vanzo, E.; Ghirardo, A.; Merl-Pham, J.; Lindermayr, C.; Heller, W.; Hauck, S. M.; Durner, J.; Schnitzler, J. P. S-nitroso-proteome in poplar leaves in response to acute ozone stress. *PLoS One* **9:**e106886; 2014.

[19] Morisse, S.; Zaffagnini, M.; Gao, X. H.; Lemaire, S. D.; Marchand, C. H. Insight into protein S-nitrosylation in Chlamydomonas reinhardtii. *Antioxid Redox Signal* **21:**1271-1284; 2014.
[20] Chaki, M.; Shekariesfahlan, A.; Ageeva, A.; Mengel, A.; von Toerne, C.; Durner, J.; Lindermayr, C. Identification of nuclear target proteins for S-nitrosylation in pathogen-treated Arabidopsis thaliana cell cultures. *Plant Sci* **238:**115-126; 2015.
[21] Vanzo, E.; Merl-Pham, J.; Velikova, V.; Ghirardo, A.; Lindermayr, C.; Hauck, S. M.; Bernhardt, J.; Riedel, K.; Durner, J.; Schnitzler, J. P. Modulation of Protein S-Nitrosylation by Isoprene Emission in Poplar. *Plant Physiol* **170:**1945-1961; 2016.
[22] Henard, C. A.; Guarnieri, M. T.; Knoshaug, E. P. The Chlorella vulgaris S-Nitrosoproteome under Nitrogen-Replete and -Deplete Conditions. *Front Bioeng Biotechnol* **4:**100; 2016.
[23] Hu, J.; Huang, X.; Chen, L.; Sun, X.; Lu, C.; Zhang, L.; Wang, Y.; Zuo, J. Site-specific nitrosoproteomic identification of endogenously S-nitrosylated proteins in Arabidopsis. *Plant Physiol* **167:**1731-1746; 2015.
[24] Parker, J.; Zhu, N.; Zhu, M.; Chen, S. Profiling thiol redox proteome using isotope tagging mass spectrometry. *J Vis Exp*; 2012.
[25] Balmant, K. M.; Parker, J.; Yoo, M. J.; Zhu, N.; Dufresne, C.; Chen, S. Redox proteomics of tomato in response to Pseudomonas syringae infection. *Hortic Res* **2:**15043; 2015.
[26] Yin, Z.; Balmant, K.; Geng, S.; Zhu, N.; Zhang, T.; Dufresne, C.; Dai, S.; Chen, S. Bicarbonate Induced Redox Proteome Changes in Arabidopsis Suspension Cells. *Front Plant Sci* **8:**58; 2017.
[27] Guo, J.; Nguyen, A. Y.; Dai, Z.; Su, D.; Gaffrey, M. J.; Moore, R. J.; Jacobs, J. M.; Monroe, M. E.; Smith, R. D.; Koppenaal, D. W.; Pakrasi, H. B.; Qian, W. J. Proteome-wide light/dark modulation of thiol oxidation in cyanobacteria revealed by quantitative site-specific redox proteomics. *Mol Cell Proteomics* **13:**3270-3285; 2014.
[28] Liu, P.; Zhang, H.; Wang, H.; Xia, Y. Identification of redox-sensitive cysteines in the Arabidopsis proteome using OxiTRAQ, a quantitative redox proteomics method. *Proteomics* **14:**750-762; 2014.
[29] Rosenwasser, S.; Graff van Creveld, S.; Schatz, D.; Malitsky, S.; Tzfadia, O.; Aharoni, A.; Levin, Y.; Gabashvili, A.; Feldmesser, E.; Vardi, A. Mapping the diatom redox-sensitive proteome provides insight into response to nitrogen stress in the marine environment. *Proc Natl Acad Sci U S A* **111:**2740-2745; 2014.
[30] Zhu, M.; Zhu, N.; Song, W. Y.; Harmon, A. C.; Assmann, S. M.; Chen, S. Thiol-based redox proteins in abscisic acid and methyl jasmonate signaling in Brassica napus guard cells. *Plant J* **78:**491-515; 2014.
[31] Liu, P.; Zhang, H.; Yu, B.; Xiong, L.; Xia, Y. Proteomic identification of early salicylate- and flg22-responsive redox-sensitive proteins in Arabidopsis. *Sci Rep* **5:**8625; 2015.
[32] Slade, W. O.; Werth, E. G.; McConnell, E. W.; Alvarez, S.; Hicks, L. M. Quantifying reversible oxidation of protein thiols in photosynthetic organisms. *J Am Soc Mass Spectrom* **26:**631-640; 2015.
[33] Muthuramalingam, M.; Matros, A.; Scheibe, R.; Mock, H. P.; Dietz, K. J. The hydrogen peroxide-sensitive proteome of the chloroplast in vitro and in vivo. *Front Plant Sci* **4:**54; 2013.
[34] Akter, S.; Huang, J.; Bodra, N.; De Smet, B.; Wahni, K.; Rombaut, D.; Pauwels, J.; Gevaert, K.; Carroll, K.; Van Breusegem, F.; Messens, J. DYn-2 Based Identification of Arabidopsis Sulfenomes. *Mol Cell Proteomics* **14:**1183-1200; 2015.
[35] Waszczak, C.; Akter, S.; Eeckhout, D.; Persiau, G.; Wahni, K.; Bodra, N.; Van Molle, I.; De Smet, B.; Vertommen, D.; Gevaert, K.; De Jaeger, G.; Van Montagu, M.; Messens, J.; Van Breusegem, F. Sulfenome mining in Arabidopsis thaliana. *Proc Natl Acad Sci U S A* **111:**11545-11550; 2014.
[36] Akter, S.; Carpentier, S.; Van Breusegem, F.; Messens, J. Identification of dimedone-trapped sulfenylated proteins in plants under stress. *Biochem Biophys Rep* **9:**106-113; 2017.

[37] Ito, H.; Iwabuchi, M.; Ogawa, K. The sugar-metabolic enzymes aldolase and triose-phosphate isomerase are targets of glutathionylation in Arabidopsis thaliana: detection using biotinylated glutathione. *Plant and Cell Physiology* **44:**655-660; 2003.
[38] Dixon, D. P.; Skipsey, M.; Grundy, N. M.; Edwards, R. Stress-induced protein S-glutathionylation in Arabidopsis. *Plant Physiol* **138:**2233-2244; 2005.
[39] Zaffagnini, M.; Bedhomme, M.; Groni, H.; Marchand, C. H.; Puppo, C.; Gontero, B.; Cassier-Chauvat, C.; Decottignies, P.; Lemaire, S. D. Glutathionylation in the photosynthetic model organism Chlamydomonas reinhardtii: a proteomic survey. *Mol Cell Proteomics* **11:**M111 014142; 2012.
[40] Aroca, A.; Serna, A.; Gotor, C.; Romero, L. C. S-sulfhydration: a cysteine posttranslational modification in plant systems. *Plant Physiol* **168:**334-342; 2015.
[41] Aroca, A.; Benito, J. M.; Gotor, C.; Romero, L. C. Persulfidation proteome reveals the regulation of protein function by hydrogen sulfide in diverse biological processes in Arabidopsis. *J Exp Bot* **68:**4915-4927; 2017.
[42] Bykova, N. V.; Rampitsch, C. Modulating protein function through reversible oxidation: Redox-mediated processes in plants revealed through proteomics. *Proteomics* **13:**579-596; 2013.
